# Epigenome editing-mediated restoration of *FBN1* expression by demethylation of CpG island shore in porcine fibroblasts

**DOI:** 10.1016/j.bbrep.2025.101973

**Published:** 2025-03-11

**Authors:** Rio Miyadai, Shiori Hinata, Yuya Amemiya, Satori Shigematsu, Kazuhiro Umeyama, Hiroshi Nagashima, Kenji Yamatoya, Jun Ohgane

**Affiliations:** a Laboratory of Genomic Function Engineering, Department of Life Sciences, School of Agriculture, Meiji University, Kawasaki, 214-8571, Kanagawa, Japan; b Meiji University International Institute for Bio-Resource Research (MUIIBR), Kawasaki, 214-8571, Kanagawa, Japan

**Keywords:** Epigenome editing, CpG island shore, FBN1, Haploinsufficiency, DNA methylation, dCas9-TET1

## Abstract

Fibrillin-1, an extracellular matrix protein encoded by the *FBN1* gene, is crucial for maintaining connective tissue integrity. Mutations in *FBN1* result in haploinsufficiency, leading to Marfan syndrome, in which the expression of functional *FBN1* is correlated with disease onset and severity. Recent studies suggest that *FBN1* expression is modulated by DNA methylation, particularly within the CpG island shores of its promoter region. In porcine models, *FBN1* mRNA levels have been found to correlate with the proportion of hypomethylated alleles in the CpG island shore region. In this study, we employed epigenome editing using the dCas9-TET1 system to induce targeted DNA demethylation within the *FBN1* CpG island shore, which became hypermethylated after a prolonged culture of porcine fetal fibroblast cells. This approach effectively reduced methylation in the targeted region, and cells expressing the dCas9-TET1 system maintained hypomethylation across multiple passages. Critically, DNA demethylation of the *FBN1* CpG island shore restored *FBN1* expression in heterozygous *FBN1* knockout fibroblasts, which developed stochastic hypermethylation after extended culture. These findings highlight the potential of DNA methylation manipulation to restore *FBN1* expression in cells with a haploinsufficient genetic background.

## Highlights

• Epigenome editing by dCas9-TET1 successfully demethylated CpG island shore of *FBN1*.
• dCas9-TET1 system maintained hypomethylation across multiple passages.
• Demethylation of CpG island shore restored *FBN1* expression in *FBN1*^*+/−*^ fibroblasts.

## 1 Introduction

DNA methylation is a mammalian epigenetic gene regulation process that occurs at cytosines in CpG dinucleotides. The methylation of CpGs in promoter regions leads to transcriptional repression [1]. CpG sequences are typically present at a lower frequency than expected in mammalian genomic DNA; however, there are regions known as CpG islands where CpGs remain at almost the same frequency as expected. A CpG island is defined as a region more than 200 bp in length with a GC content of greater than 50 % and a relative CpG frequency greater than 0.6 [2]. CpG islands are located within promoter regions, including transcription start sites. The CpG island shore, which is the boundary between the CpG island and the adjacent region with low CpG frequency, contains many tissue-dependent differentially methylated regions (T-DMRs) [3,4]. The DNA methylation status of T-DMRs varies depending on the tissue or cell type and is important for tissue/cell-type-dependent gene expression.

T-DMRs are present in the promoters of tissue- and cell-type-specific genes and regulate their expression [3,5]. Sodium bisulfite sequencing is a conventional DNA methylation analysis method that identifies DNA methylation patterns in each sequenced fragment that can be recognized as a single allele (cell) in a tissue or cell population. Based on this concept, we previously focused on transcriptionally active DNA hypomethylated alleles (hypo-alleles), in which the methylated CpG ratio was less than 25 % [6]. Furthermore, we reported that in populations consisting of multiple cell types, such as tissues and primary cultured cells, the proportion of hypomethylated alleles (hypo-allele ratio) was correlated with the proportion of cells expressing the corresponding gene [6]. This suggests that changes in the hypo-allele ratio reflect changes in the proportion of expressible cells and that the hypo-allele ratio in T-DMRs can be an indicator of the proportion of cells expressing the analyzed genes in cell populations.

The *Fibrillin-1* gene (*FBN1*) encodes a crucial extracellular matrix protein that is widely distributed in connective tissues and plays a significant role in the pathogenesis of Marfan syndrome [7,8]. This genetic disorder follows an autosomal dominant inheritance pattern, often resulting from mutations in one *FBN1* allele [9], leading to haploinsufficiency [7,10]. Clinical studies have revealed that individuals with reduced *FBN1* expression are at higher risk for specific manifestations of Marfan syndrome, including ocular, skeletal, and cardiovascular complications [11]. Consequently, the quantity of functional Fibrillin-1 protein, determined by *FBN1* expression levels, is closely linked to the onset and progression of Marfan syndrome. Our previous study has highlighted the importance of epigenetic regulation in *FBN1* expression. Investigations using a porcine model demonstrated that DNA methylation patterns in the *FBN1* promoter region, particularly in the CpG island shore, correlate with *FBN1* mRNA levels [12,13]. We also found through comparative genomic analysis that the porcine *FBN1* CpG island shore exhibits higher sequence homology to its human counterpart than to the mouse ortholog, including similarities in the number of methylated CpG dinucleotides (Supplementary Fig. 1). These results suggest that *FBN1* expression is regulated by variable changes in epigenetic modifications in patients with Marfan syndrome.

Recently, epigenome editing based on CRISPR/Cas9 technology has been used to directly fuse or indirectly assemble epigenetic effectors into nuclease-inactive Cas9 (dCas9) together with guide RNA (gRNA) directed to target regions [14,15]. Our data also indicate that induced epigenome editing using Dnmt3a/3l resulted in DNA hypermethylation of the target gene *Runx2*, which was correlated with reduced expression [16]. Moreover, extended cultures of primary porcine fetal fibroblast cells exhibited stochastic DNA methylation changes in both the hyper- and hypomethylation directions in the T-DMR of *FBN1* CpG island shore, although DNA hypermethylation in the *FBN1* CpG island shore was more frequent than DNA hypomethylation [12]. These data suggest that DNA methylation levels and/or hypo-allele ratios are associated with *FBN1* mRNA levels.

Our previous data indicate that stochastic DNA hypermethylation during prolonged porcine fibroblast culture correlated with reduced expression of *FBN1,* most likely resulting from a decrease in the number of *FBN1*-expressing cells under heterozygous knockout conditions [12]. Subsequently, whether the inhibition of DNA hypermethylation can lead to the recovery of *FBN1* expression levels even after prolonged cell culture remains unclear. Therefore, in the present study, we aimed to introduce targeted DNA demethylation within the *FBN1* CpG island shore, which becomes hypermethylated after a prolonged culture of porcine fetal fibroblast cells, by epigenome editing using the dCas-TET1 system.

## 2 Materials and methods

### 2.1 Construction of plasmids for targeted demethylation

The dCas9-SunTag-TET system all-in-one vector for the expression of dCas9/tetra-GCN4 linked to scFv/GFP/TET1CD by the P2A peptide under the control of the CAG promoter and gRNA under the U6 promoter (pPlat-TET-gRNA2 Addgene plasmid 82559) [15] were generated by inserting the target sequence (Supplementary Table S1) into *Afl*II site using T4 DNA Ligase (Promega co., Madison, WI, USA). Five inserts for gRNAs, gRNA3 to gRNA7 were designed using CRISPRdirect (https://crispr. dbcls. jp/) and were purchased as single-stranded oligonucleotides bearing cohesive ends for *Afl*II (Eurofins Genomics Corp., Tokyo, Japan). Equal amounts of complementary strands were mixed, heat-treated to form double strands, and treated with T4 polynucleotide kinase (Takara Bio Inc., Shiga, Japan) before ligation into pPlat-TET-gRNA2.

### 2.2 Porcine fibroblast cell culture and transfection

The primary culture of porcine fibroblasts was performed as previously described [12,17]. Wild-type porcine fibroblasts W239_1 and W241_1, along with *FBN1*^*+/−*^ fibroblasts W241_2, were used in this study. The *FBN1*^*+/−*^ fibroblasts show significantly lower FBN1 mRNA expression compared to wild-type cells (Supplementary Fig. 2) [12]. Cells were cultured in alpha-modified Eagle's minimum essential medium (FUJIFILM Wako Chemical Co., Kanagawa, Japan) supplemented with 15 % fetal bovine serum (Thermo Fisher Scientific K.K., Tokyo, Japan) and penicillin-streptomycin solution (Thermo Fisher Scientific) with type I collagen (Nitta gelatin Inc., Osaka, Japan) coated dish at 5 % CO_2_, 37 °C. Cells were transfected using Neon transfection system 100-μL Kit (Thermo Fisher Scientific). Transfected cells were cultured in 0.5 mg/ml G418 (Thermo Fisher Scientific) for two weeks, and stable transfectants were obtained.

### 2.3 DNA methylation analysis

Bisulfite sequencing analysis was performed as previously described [18]. Bisulfite treated porcine *FBN1* CpG island shore region was PCR amplified using KOD One PCR master mix (TOYOBO) and specific primers (FBN1_Bis_F: 5′-AGTTTTAATGTGAGTTGGATAAAAGGA-3′, FBN1_Bis_R: 5′-AAAAACTATACCACCTACACCAAAAA-3′) and dA overhang were added using TArget Clone™ -Plus- (TOYOBO). The amplified fragments were ligated into pGEM-T Easy vectors (#A1360, Promega). A minimum of ten clones were selected, and the vector was isothermally amplified using phi29 DNA Polymerase (New England Biolabs Japan Inc., Tokyo, Japan). Sequencing was performed using an Applied Biosystems 3130xL system (Thermo Fisher Scientific).

### 2.4 RT-PCR analysis

Total RNA was extracted using a FastGene RNA Basic Kit (NIPPON Genetics Co. Ltd., Toyo, Japan). First-strand cDNA synthesis with transcript-specific primers was performed using Superscript III First-Strand Synthesis System (Thermo Fisher Scientific). RT-PCR was performed using TaKaRa LA Taq (Takara) with specific primers for FBN1 mRNA (FBN1_RT_F1: 5′-ATGTCCCTATGGTAGTTCCTGAGA-3′, FBN1_RT_R1:5′-AGTTGGAATCCTTTGTTGCACT-3′), and GAPDH (GAPDH_RT_F: 5′-ACCACAGTCCATGCCATCAC-3′, GAPDH_RT_R: 5′-TCCACCACCCTGTTGCTGTA-3′). RT-PCR was performed using the following conditions: 95 °C for 5 min; 26 cycles (FBN1 mRNA), or 23 cycles (GAPDH) of 95 °C for 30 s, 60 °C for 30 s, and 72 °C for 1 min; final extension 72 °C for 10 min. Band intensities were analyzed using the ImageJ software (National Institutes of Health, Bethesda, MD, USA).

We performed cDNA synthesis for Quantitative PCR using ReverTra Ace qPCR RT Master Mix with gDNA Remover (TOYOBO Co. Ltd., Osaka, Japan). qPCR was performed using the KOD SYBR qPCR Mix (TOYOBO) on a StepOnePlus Real-Time PCR System (Thermo Fisher Scientific). The specific primers used for the qPCR were as follows:

*β-actin*_F: 5′-GATCTGGCACCACACCTTCTACAAC-3′, *β-actin*_R: 5′- GCCAGAGGCGTACAGGGACAGCAC-3′, *FBN1*_F: 5′-TCTGTCCCTATGGTAGTTCCTGAGAG-3′, *FBN1*_R: 5′-TCCTTTGTTGCACTCACACCGGTAGC-3′, *TIMP1*_F: *5*′*- TGTGGGAGCCCCAGAGTTCAACCAG-3*′*, TIMP1*_R: *5*′*- AGCCACAAAACTGCAGGTGGTGATG-3*′*, TIMP2*_F: *5*′*-GTACCAGATGGGCTGTGAGTGCAAG-3*′*, TIMP2*_R: 5′-AGTCTGGACTCTCGGAGGCTCTCTG-3′. Relative gene expression levels were determined using the comparative delta-delta Ct method using *β-actin* as a housekeeping gene.

### 2.5 Construction of plasmids for luciferase reporter assay

The promoter region, including CpG island to 5′UTR of porcine *FBN1* (−607 to +767 bp relative to transcription start site) and CpG island shore region (−878 to −639 bp), were cloned into CpG-free reporter vector: pCpGL [19]. The *in vitro* methylation of the vector was performed using CpG methyltransferase (New England Biolabs) according to the manufacturer's protocol. Each of the pCpGL vectors (2 μg) and Renilla luciferase control vector pRL-TK (100 ng) were transiently transfected into 2x10^5^ cells of wild-type porcine fibroblast. The lysates were analyzed using the Dual-Luciferase Reporter Assay System (Promega).

### 2.6 Statistical analysis

Data are represented as means ± Standard Deviation (SD). The statistical significance of the experiments was determined using Student's t-test with Welch's correction.

## 3 Results

### 3.1 DNA methylation analysis of the FBN1 promoter region after targeted DNA demethylation

**Fig. 1:**
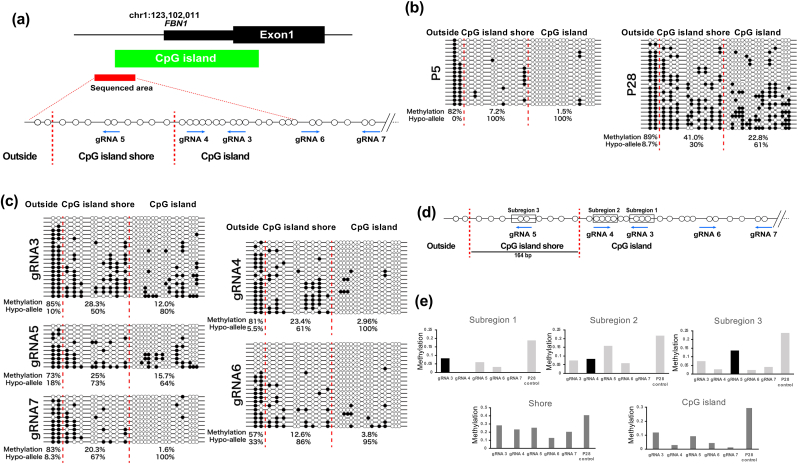
DNA methylation analysis of the FBN1 promoter region in late-passage WT porcine fibroblast cells stably transfected with the dCAs9-Sun Tag-TET system with various gRNAs. (a) FBN1 promoter regions were divided into three sub-regions by red dotted lines based on their respective DNA methylation statuses (Outside, the CpG island shore, and the CpG island). White and black dots indicate unmethylated and methylated CpGs. In the porcine FBN1 genomic region, the CpG island and sequenced area are indicated in green and red, respectively. CpG positions and gRNA target sites are shown as circles and blue arrows, respectively. (b) Bisulfite sequencing results of the FBN1 promoter region in fibroblast cells of passage 5 (P5) and passage 28 (P28). During the prolonged passage of porcine fibroblast cells, the CpG island shore exhibited increased DNA methylation levels from 7.2 % to 41.0 % and decreased Hypo-allele ratios from 100 % to 30 %. In contrast, the CpG island and the outside area did not show any significant changes in both DNA methylation levels and Hypo-allele ratios. (c) Bisulfite sequencing results of late-passage fibroblast cells transfected with the dCas9- SunTag-TET system using various gRNAs. The rates of methylated CpG and hypo-alleles for each subregion are provided below the lollipop diagrams, respectively. (d) The schematic diagram indicates three subregions (boxes) targeted by gRNAs (blue arrows) and overlapping CpGs (white circles) in the boxes within and adjacent to the CpG island shore. The scale bar indicates the length of CpG island shore 164 bp. (e) DNA methylation rates of the subregions, CpG island, and shore. Black bars indicate the gRNAs that overlap with the subregions analyzed. Cells were transfected at P28 and analyzed at P30.

In our previous report [12], we assessed the CpG island shore and the region between invariably methylated and unmethylated regions and designed gRNAs (Fig. 1a). The effective range of the pPlat-TET-gRNA2 system is 200 bp to 1 kbp [15]. Therefore, five gRNAs (gRNA3 to gRNA7) were designed in a 400-bp range, including CpG island shore and adjacent CpG island. Of the five gRNAs, one was located within the CpG island shore (gRNA5), and the other four were in the CpG island (gRNAs 3, 4, 6, and 7).

To test the demethylation efficiency of gRNA-targeted ten-eleven hydroxylase (TET1), we transfected the vectors into late-passage cells (P28), which confirmed the accumulation of CpG methylation in the *FBN1* promoter region (Fig. 1b). All five gRNA expression vectors induced significant DNA demethylation in the CpG islands and shore regions (Fig. 1c). Notably, gRNAs 4, 6, and 7 showed efficient DNA demethylation in both the CpG island and shore regions. Our careful investigation of DNA demethylation efficiency at each CpG in subregions 1–3 indicated that CpGs within the gRNA-binding sites tended to be resistant to DNA demethylation, probably due to the partial suppression of TET1 activity, and CpGs adjacent to the gRNA-binding sites were more effectively demethylated than gRNA-overlapping CpGs (Fig. 1e upper panel). Additionally, the CpG island shore region exhibited higher resistance to DNA demethylation than the CpG island region (Fig. 1e, lower panel).

### 3.2 Analysis of DNA methylation changes during passages of transfectant cells

**Fig. 2:**
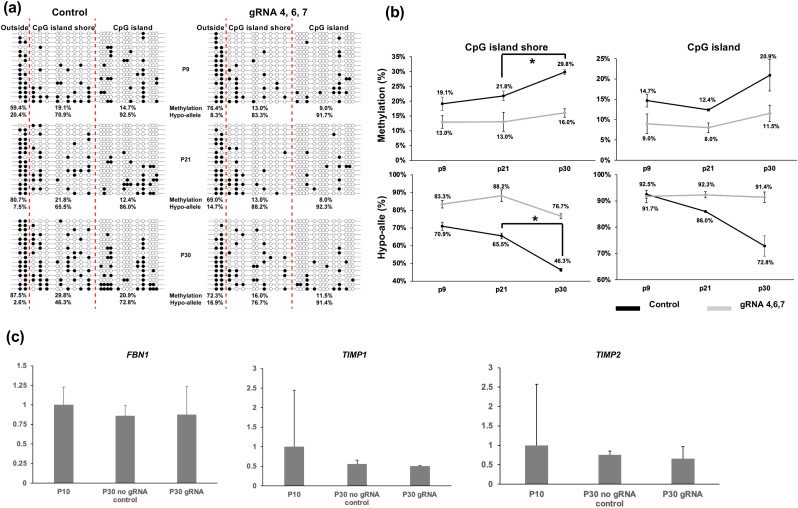
DNA methylation analysis of WT cells stably transfected with the Sun Tag system containing mixture of gRNAs 4, 6, and 7 during passage progression, and expression analysis of genes associated with aortic aneurysm by qRT-PCR. (a) Lollipop diagram with rates of methylated CpG and Hypo-alleles of P9, P21, and P30. (b) Changes of methylated CpGs and Hypo-alleles. Asterisks indicate significant differences (p < 0.05). (c) Wild-type late-passage porcine fibroblasts transfected with a sun-tag system containing a mixture of gRNAs 4, 6, and 7 or control vector without gRNA. Early passage cells for control were at P10. Gene expression data are presented as means ± standard deviation, derived from three independent biological replicates using β-actin as the housekeeping gene.

To test the long-term efficacy of the pPlat-TET-gRNA2 system for DNA demethylation on the *FBN1* CpG island shore, we co-transfected the three most efficient gRNA-expressing vectors (gRNAs 4, 6, and 7) into early passage WT fibroblasts (P8) and established a stable transfectant. The resulting cells contained hypo- and hypermethylated alleles at P30, at levels comparable to those at P9. However, cells with gRNA-expressing vectors inhibited the increase in DNA methylation rate and decreased the hypo-allele ratio compared to control cells between P21 and P30 (Fig. 2b).

### 3.3 Effect of FBN1 CpG island shore demethylation on expressions of Marfan syndrome-associated genes

Since extracellular matrix maintenance is a complex process involving the synthesis, assembly, and degradation of various proteins, we investigated the expression of tissue inhibitors of the metalloproteinases TIMP-1 and TIMP-2, which are essential for elastic fiber integrity. Patients with aortic aneurysms frequently exhibit variations in the coding sequences of these proteins [20]. Demethylation of the *FBN1* CpG island shore tended to restore *FBN1* mRNA expression levels, which decreased after multiple passages; however, the difference was not statistically significant (Fig. 2c). *TIMP-1* and *TIMP-2* expression levels did not differ significantly between the cells transfected with the gRNA-expressing vector and those transfected with the control vector. The genes which affect the assembly of elastic fibers (FBLN4, FBLN5, MFAP4) were also investigated and the differences were not statistically significant (Supplementary Fig. 3).

### 3.4 Effect of FBN1 CpG island shore demethylation in FBN1^+/−^ fibroblast

**Fig. 3:**
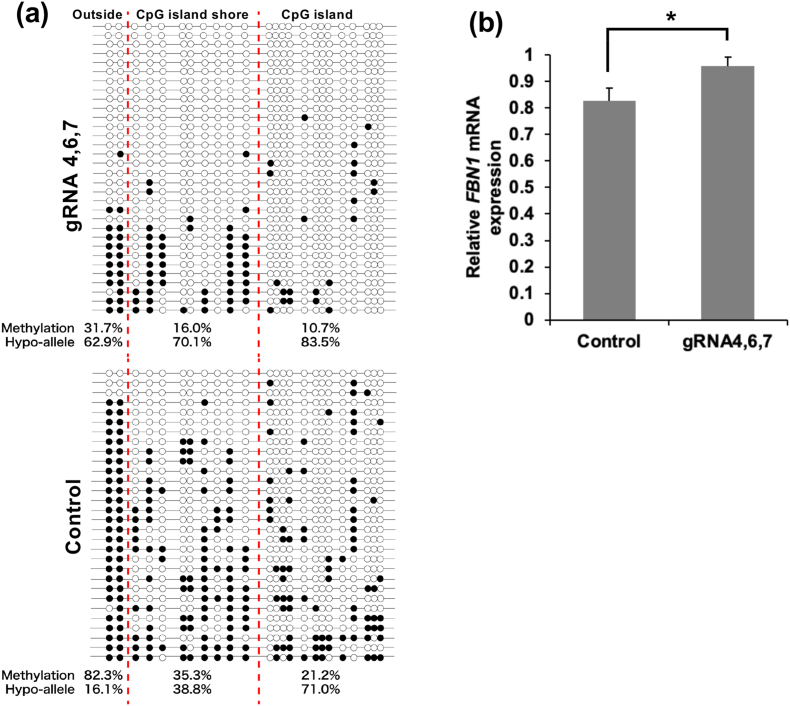
Effect of targeted DNA demethylation of the CpG island shore on FBN1 expression in FBN1 ± fibroblast cells. (a) FBN1 ± fibroblast cells were transfected with the Sun Tag system containing a mixture of gRNAs 4, 6, and 7. DNA methylation levels and Hypo-allele ratios of stably transfected and control cells were shown below the lollipop diagrams. (b) RT-PCR analysis of the FBN1 mRNA expression in FBN1 ± fibroblast cells. Values are the mean ± SD from three biological replicates. Asterisk indicate a significant difference (p < 0.05).

To investigate the effects of DNA demethylation on *FBN1* mRNA expression in Marfan-comparable heterozygous knockout cells, we utilized P23 *FBN1*^*+/−*^ fibroblasts transfected with vectors containing gRNAs 4, 6, and 7. DNA methylation analysis revealed that the dCas9-SunTag-TET system effectively demethylated the entire sequenced region, including the areas outside the CpG island shore (Fig. 3a). Cells transfected with gRNA 4, 6, and 7 vectors exhibited significantly higher *FBN1* expression than control cells (Fig. 3b). This suggests that DNA demethylation of the promoter region successfully restored mRNA transcription levels in *FBN1*-heterozygous knockout cells of the same genotype as patients with Marfan syndrome.

### 3.5 Function of CpG island shore in FBN1 promoter

**Fig. 4:**
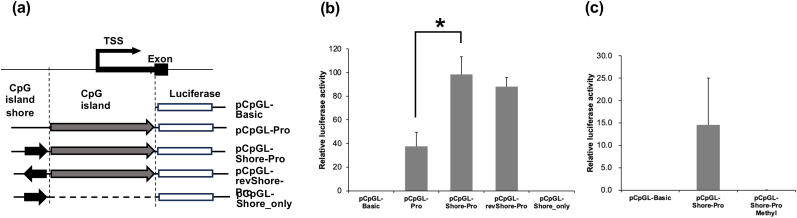
Effect of CpG island shore reversal in reporter assay of FBN1 promoter region. (a) Schematic diagram of vector construction. Porcine fibroblast cells were transiently transfected with the FBN1 promoter reporter plasmids, and the Firefly luciferase activity of each plasmid was normalized to the Renilla luciferase activity from the co-transfected plasmid. (b) The reporter assay results are presented as a ratio of Firefly to Renilla luciferase activity. (C) Luciferase reporter assay of the CpG-methylated FBN1 promoter region. Values are the mean ± SD of three independent samples. Asterisk indicate a significant difference (p < 0.05).

Furthermore, we employed reporter gene assays to elucidate whether the CpG island shore region is involved in *FBN1* gene regulation. Depletion of the CpG island shore significantly reduced the promoter activity to below half of that observed in promoter with the CpG island shore. In contrast, reversing the CpG island shore direction did not cause a significant decrease in promoter activity (Fig. 4b), suggesting that the main function of the CpG island shore is to increase the *FBN1* promoter activity as an enhancer. *In vitro* CpG methylation of the *FBN1* promoter region abolished promoter activity (Fig. 4c), indicating that methylation of the *FBN1* promoter region, including the CpG island shore, inactivated the *FBN1* gene.

## 4 Discussion

We previously found that DNA methylation status on the CpG island shore correlated with *FBN1* expression level [13]. In this study, we aimed to see whether DNA methylation directly affect the *FBN1* expression, and targeted DNA demethylation of the CpG island shore via epigenome editing increased *FBN1* expression. In addition, careful investigation of the effect of epigenome editing on individual CpGs indicated that CpGs overlapping with gRNA target sites were less effective for DNA demethylation than neighboring CpGs, probably due to steric hindrance caused by binding of the dCas protein to the gRNA target sites, and the combination of multiple gRNAs could overcome the ineffectiveness of DNA demethylation of the gRNA-overlapping CpGs. Co-transfection of the three gRNA-expressing vectors into primary cultured fibroblasts induced the most efficient targeted DNA demethylation of the *FBN1* promoter region, including the CpG island shore. In summary, we established a method to suppress stochastic DNA hypermethylation of the porcine *FBN1* CpG island shore during prolonged culture of primary fibroblasts.

In the porcine *FBN1* promoter region, the CpG island shore contains a T-DMR that is hypomethylated in *FBN1*-expressing tissues or cell types such as fibroblast cells [13]. In addition to tissue/cell type-dependent DNA methylation, stochastic changes in DNA methylation status have been previously observed on the CpG island shore during the extensive passage of fibroblast cells [12]. In humans, heterozygous individuals exhibit a spectrum of Marfan syndrome phenotypes at and after the onset of the disease for unidentified reasons. In the present study, epigenome editing for DNA demethylation worked efficiently by introducing multiple gRNAs targeting the porcine *FBN1* CpG island shore region. Using this system, porcine fibroblast cells with an *FBN1* haploinsufficient genetic background [13,17] could overcome the DNA hypermethylation trend and effectively restore *FBN1* mRNA levels to some extent. These findings, together with those of our previous report [12], indicate that stochastic DNA hypermethylation is the cause of *FBN1* downregulation in the normal allele, resulting in a decrease in the number of cells expressing *FBN1*. Thus, targeted DNA demethylation on the *FBN1* CpG island shore increases the hypo allele ratio, increasing the number of *FBN1*-expressing cells.

Marfan syndrome caused by haploinsufficiency is likely associated with expression levels from the normal *FBN1* allele [11]. Our data indicate that induced DNA demethylation of the *FBN1* CpG island shore could restore transcription from the normal allele in porcine cells. DNA sequence comparisons between humans and pigs indicate that the porcine *FBN1* promoter sequence is highly homologous to human *FBN1*. This finding led us to hypothesize that human *FBN1* is also regulated by DNA methylation and that stochastic DNA hypermethylation is involved in the onset of Marfan syndrome. Although epigenome editing in patients with Marfan syndrome is not realistic, inhibiting stochastic DNA hypermethylation progression may support prevention and treatment in the future.

Our porcine model of Marfan syndrome with a haploinsufficient genetic background strongly indicates that stochastic DNA hypermethylation causes Marfan syndrome onset and progression [12,17]. While many diseases exhibit a wide spectrum of phenotypes associated with haploinsufficiency, these variable presentations cannot be sufficiently explained by classical genetics, which typically emphasizes simple DNA sequence variations within genes implicated in haploinsufficiency-related disorders. Approximately half of the mammalian genes contain CpG islands/shores in their promoter regions, and many haploinsufficiency disease-responsible genes also fall into the same criteria. In this study, targeted DNA demethylation of the *FBN1* CpG island shore successfully restored *FBN1* expression in primary extended-culture cells with a heterozygous knockout for *FBN1*. Thus, the stochastic outcome of DNA hypermethylation is likely to be a cause of the depletion of disease-responsible genes in individuals with a haploinsufficient genetic background. Consequently, the avoidance of DNA hypermethylation in individuals with a haploinsufficient genetic background likely results in the prevention and reduction of disease onset or progression.

## CRediT authorship contribution statement

**Rio Miyadai:** Investigation, Data curation. **Shiori Hinata:** Investigation, Data curation. **Yuya Amemiya:** Investigation, Data curation. **Satori Shigematsu:** Investigation, Data curation. **Kazuhiro Umeyama:** Resources. **Hiroshi Nagashima:** Resources, Conceptualization. **Kenji Yamatoya:** Writing – review & editing, Writing – original draft, Supervision, Investigation, Data curation. **Jun Ohgane:** Writing – review & editing, Writing – original draft, Supervision, Funding acquisition, Conceptualization.

## Data statement

The data supporting the findings of this study are available within the article.

## Funding

This work was supported by JSPS KAKENHI Grant Number JP21H04755 to J.O. The studies on Marfan Syndrome model porcine cells described in this paper received financial support from the 10.13039/100014424Meiji University Institute for Bio-Resource Research (MUIIBR), and Leading Advanced Project for Medical Innovation (LEAP), 10.13039/100009619AMED.

## Declaration of competing interest

The authors declare that they have no known competing financial interests or personal relationships that could have appeared to influence the work reported in this paper.

## Glossary

CpG: A shorthand notation for 5′-C-phosphate-G-3′, which represents a specific DNA sequence where cytosine (C) is followed by guanine (G) with a phosphodiester bond in between.
