# Supplementary material for: Epigenome editing-mediated restoration of FBN1 expression by demethylation of CpG island shore in porcine fibroblasts
Source: Biochem Biophys Rep. 2025 Mar 11;42:101973. doi: 10.1016/j.bbrep.2025.101973 (PMC11932662; doi:10.1016/j.bbrep.2025.101973)
Supplement: Multimedia component 1 [file mmc1.docx]

| Table S1. ssDNAs used for gRNA vector construction. | | | |
| --- | --- | --- | --- |
| DNA sets for gRNAs | | Sequence |  |
| gRNA3-F | 5'-ttaaggctggagaagcgacgcgtccc-3' | | |
| gRNA3-R | 5'-ttaagggacgcgtcgcttctccagcc-3' | | |
| gRNA4-F | 5'-ttaaggaggccgcggcgcggagacac-3' | | |
| gRNA4-R | 5'-ttaagtgtctccgcgccgcggcctcc-3' | | |
| gRNA5-F | 5'-ttaagggggctggggtctagcggcgc-3' | | |
| gRNA5-R | 5'-ttaagcgccgctagaccccagccccc-3' | | |
| gRNA6-F | 5'-ttaagggtggcacagctttccgcgcc-3' | | |
| gRNA6-R | 5'-ttaaggcgcggaaagctgtgccaccc-3' | | |
| gRNA7-F | 5'-ttaagtagcggtttcgcccgctctac-3' | | |
| gRNA7-R | 5'-ttaagtagagcgggcgaaaccgctac-3' | | |
