# Supplementary material for: Epigenome editing-mediated restoration of FBN1 expression by demethylation of CpG island shore in porcine fibroblasts
Source: Biochem Biophys Rep. 2025 Mar 11;42:101973. doi: 10.1016/j.bbrep.2025.101973 (PMC11932662; doi:10.1016/j.bbrep.2025.101973)
Supplement: Multimedia component 2 [file mmc2.pdf]

Supplemental figure review only

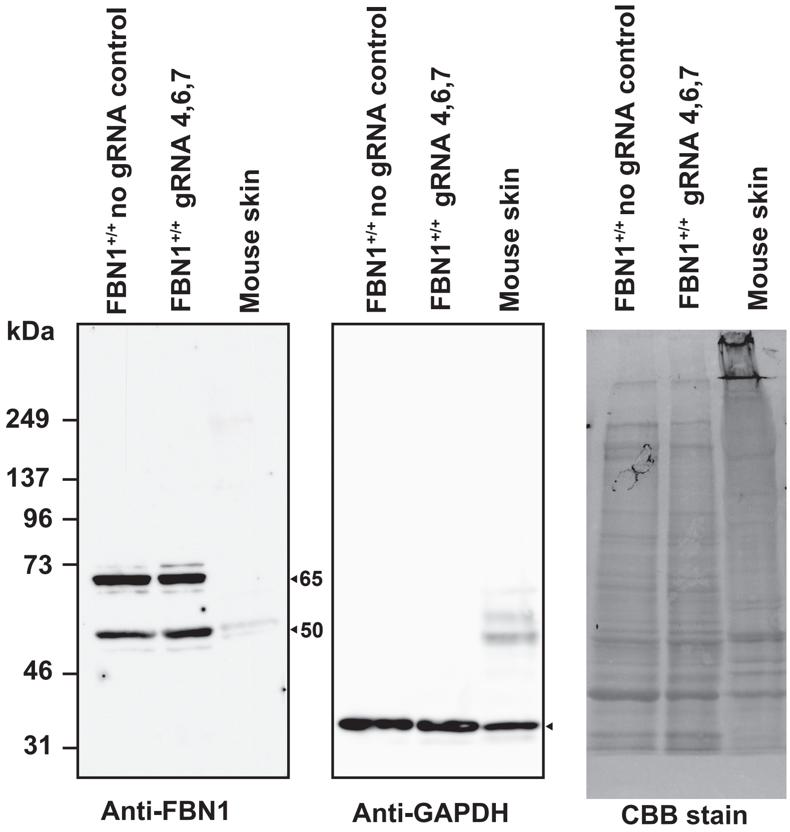

Supplemental figure review only: SDS-PAGE analysis of protein extracts from FBN1$^{+/+}$pig fibroblasts and mouse skin.

Protein extracts were prepared from pig fibroblasts (cultured for one week) and mouse skin (FBN1 positive control) using 2X SDS sample buffer (100 mM Tris-HCl [pH 6.8], 4% SDS, 200 mM dithiothreitol [DTT], 20% [v/v] glycerol). Samples (10 µg protein/lane) were separated on a 7.5% SDS-polyacrylamide gel and blotted on PVDF membrane. PVDF membrane was stained with Coomassie Brilliant Blue R-250 to visualize protein bands. bands detected by anti-FBN1 antibody and GAPDH were indicated by arrowheads.

This study was approved and conducted according to the guidelines for the care and use of laboratory animals of the Meiji University.
