# Supplementary material for: Epigenome editing-mediated restoration of FBN1 expression by demethylation of CpG island shore in porcine fibroblasts
Source: Biochem Biophys Rep. 2025 Mar 11;42:101973. doi: 10.1016/j.bbrep.2025.101973 (PMC11932662; doi:10.1016/j.bbrep.2025.101973)
Supplement: Multimedia component 3 [file mmc3.pdf]

Supplemental Figure 1

```
Human    ACAGGCCCCTGGGTTGGACACCGACTTAGAGA----GAGCACCCTGGATTCAGGCCTGGGTGGCTTGAATTGAGC
Pig      ACAGGCCTCTGGGTTGGAATCCCACTCAGACTCTTCGATCATCTTGCGTTC--AGCCTGGGTGCTTGAACCGAAA
Mouse    ACAGGCCCCCACATCACAGACTGAGACTTTCGCAGCCTCTTCATTTAGCCT--AGGTGAGTAGCTTGACCTGAGC

cons     ******* *    *   *  *  *                    *              *  ******   **


Human    CTCAGAAGAGCCGCGTCTGGAGTGGGCTCTCGACACCCAGGGCAAGTGGGGGCGGCAGAGCCCTCTCCTCGGTCG
Pig      TTCAGATGAGCCTCGCCTCCAGTGGGTCT--------CGTTGCCAGTGTCCAAGGCAGAGCCCCCACCCCCGCCG
Mouse    CTCGGGAGAATTGCTCCTCCAGCGGGCTCCTGACA-----------------CTGGCATGCCCCTTTCGCCGCCG

cons      ** *  **    *  **  ** ***                            *    ****    * * * **


Human    GCA--CAGCAGCCTCTGCCGCGGTC----CCGGCCTGCGACGCGCCCAGTCTTAGCCTCCCGGCCTCCGGCGTCT
Pig      CTAGACCCCAGCCCCTGCGGTCACC----CTGGCCCACGACCTGCCCTGTCCCGGCCTCCAGGCCTTGGGCCTCC
Mouse    CTA--ACCTGTTGGCTGGCTCAGTGGTGGTGGCCTCACCACAAGCCCTGCCTGAGCCTCGGAGCCTACAATTTCT

cons       *           ***              * *   * **  **** * *   *****   ****      **

Human    GCTGAGTGTCCGGCGGGAGAGGCGCAGGGAGCGCGCTACCGGGAGGCGCGGGCAGCGGGGACTGGTTTTCTCTCG
Pig      GCTCTGTGTCTGGGAGGC-----ACAGTGAGGCCGCGGCGCGGAGACACGGGCAACCTGGACGCGTCGCTTCTCC
Mouse    CCTCCCAGCCAGACAGCA----------GAAGACTGGGCTGAGAAGCGAGGGCCACTGCCACAGAGCCCCTTTCC

cons      **    * * *   *            **   *    *   **  *  ****  *    **        * **


Human    GG---------CCAGGGCCTCCGGGGCAACCGTCTCCAGCGCGCATTCTTGGTGCAGGTGGAACA
Pig      AGCAGGGTCCGCTGGGGCCTCCGGAGCCACAGTTTCTAGCGCGCGTTCTTGGTGCAGGTGGCACA
Mouse    AGCCAGGGCCTCCTGGGCCTCGTTGGCCAAGGTGTCCAGCATGCGTTCTCCGCGCAGGTGGCCCA

cons      *         *  *******    ** *  ** ** ***  ** ****  * ********  **
```

Supplemental Figure 1. Alignment of Human, Pig, and Mouse *FBN1* CpG island shore sequences. CpG dinucleotides are highlighted in yellow. Asterisks (*) indicate bases that are fully conserved across all three species (Human, Pig, and Mouse). Mice possess a significantly lower number of CpGs compared to humans and pigs.

Supplemental Figure 2

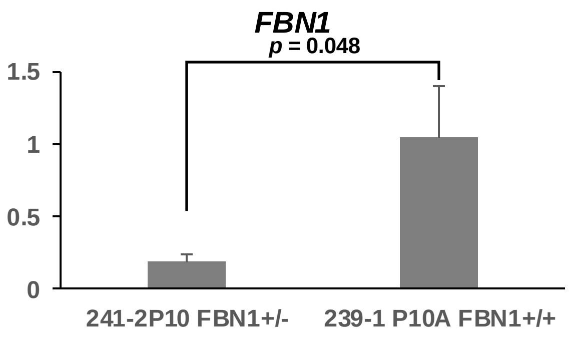

Supplemental Figure 2. The expression of *FBN1* in WT and *FBN1*$^{+/-}$ fibroblasts . Gene expression data are presented as means ± standard deviation, derived from three independent biological replicates using *β-actin* and *PPIA* as the housekeeping gene.

Supplemental Figure 3

(a)

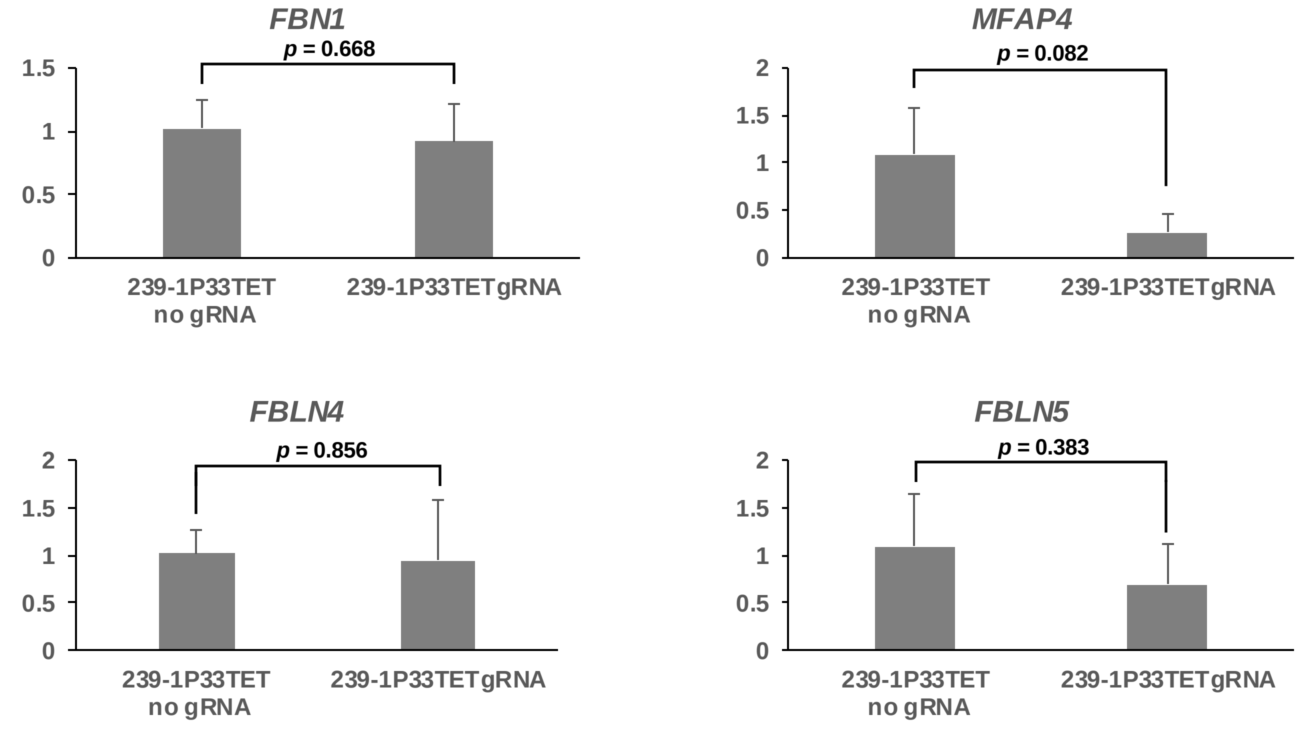

Supplemental Figure 3. The expression of genes related to elastic fiber deficiency. Gene expression data are presented as means ± standard deviation, derived from three independent biological replicates using *β-actin* and *PPIA* as the housekeeping gene.

Supplemental Materials and Methods

DNA sequence alignment for FBN1 CpG island shore

Human, Pig, and Mouse *FBN1* CpG island shore sequence alignment was created using T-coffee version 11.00 (Notredame *et al.*, J Mol Biol, 2000).

Quantitative PCR analysis for elasticfiber associated genes

The cDNAs were synthesized using SuperPrep II Cell Lysis & RT Kit for qPCR (TOYOBO Co. Ltd., Osaka, Japan). The qPCR was performed using the KOD SYBR qPCR Mix on a StepOnePlus Real-Time PCR System. The specific primers used for the qPCR were as follows: beta-2-macroglobulin (B2M)_R: 5′-CAGTCAGACCTGTCTTTCAGCAAGG-3′, B2M_R: 5′- CTCTGTGATGCCGGTTAGTGGTCTC-3′, peptidylprolyl isomerase A (PPIA)_F: 5′-GGCAAATGCTGGCCCCAACACAAAC-3′, , PPIA _R: 5′-TTGCTGGTCTTGCCATTCCTGGACC-3′, microfibril-associated glycoprotein 4 (MFAP4)_F: 5′-ATAGACGGCGTGTACCTCATCTACC
-3′, MFAP4_R: 5′-GAGAAGTCGTGGTACTTGGCGAAGG-3′, fibulin4 (FBLN4)_F: 5′-GGCCTGCAAGGGGGAAATGAAATG-3′, FBLN4 _R: 5′-TTGGTAGGAGCCAGGCAGGTTGTG-3′, fibulin5 (FBLN5) _F: 5′- ACCATTCTGGCGCTCTGTCTTCC-3′, FBLN5 _R: 5′- CCTCGGTACACTGGGTTCGTTCG-3′. The comparative delta-delta Ct method (Silver *et al.*, BMC Mol Biol, 2006) was conducted, and the two most stable genes, β-actin and PPIA, were used as housekeeping genes.

References

C. Notredame, D.G. Higgins, J. Heringa, T-Coffee: A novel method for fast and accurate multiple sequence alignment, J Mol Biol 302 (2000) 205-217. https://doi.org/ 10.1006/jmbi.2000.4042.

N. Silver, S. Best, J. Jiang, S.L. Thein, Selection of housekeeping genes for gene expression studies in human reticulocytes using real-time PCR, BMC Mol Biol 7 (2006) 33. https://doi.org/ 10.1186/1471-2199-7-33.
